# Extruded Enzyme-Added Corn Improves the Growth Performance, Intestinal Function, and Microbiome of Weaning Piglets

**DOI:** 10.3390/ani12081002

**Published:** 2022-04-13

**Authors:** Dan Zhu, Lianqiang Che, Bing Yu, Daiwen Chen

**Affiliations:** Key Laboratory for Animal Disease-Resistance Nutrition of China Ministry of Education, Institute of Animal Nutrition, Sichuan Agricultural University, Chengdu 611130, China; zhud@tongwei.com (D.Z.); che.lianqiang@sicau.edu.cn (L.C.); ybingtian@163.com (B.Y.)

**Keywords:** weaning piglet, extruded corn, enzyme addition, amylase, growth performance, intestinal function, microbiome

## Abstract

**Simple Summary:**

Energy feeds such as corn, wheat, and barley usually contain large amounts of raw starch, which is generally required to be extruded for animals. In addition, amylase preparations are widely added into the diet to degrade raw starch and promote its digestion in livestock animals. The in vitro degradation of corn starch could be a strategy for improving the starch digestion, as it is unknown whether the starch structure and its in vivo digestion could be improved by adding amylase during extrusion, regulating the extrusion moisture content. In this study, we aimed to investigate the effects of extruded corn with added amylase under different moisture conditions on the growth performance, intestinal function, and microbiome of weaning piglets.

**Abstract:**

The objective of this study was to evaluate the effects of extruded corn with added amylase under different moisture conditions on the growth performance, intestinal function, and microbiome of weaning piglets. Fourty-eight 24-day-old weaning piglets (Duroc × Landrace × Yorkshire, weaned at 22 ± 1 d) with an initial body weight of 6.76 ± 0.15 kg were randomly assigned to one of four dietary treatments with six replicates per treatment and two pigs per replicate: (1) NL (adding 7.5% water before corn extrusion, negative treatment with low moisture); (2) NH (adding 15.0% water before corn extrusion, negative treatment with high moisture); (3) PL (adding 7.5% water and 4 kg/t α-amylase before corn extrusion, positive treatment with low moisture); and (4) PH (adding 15% water and 4 kg/t α-amylase before corn extrusion, positive treatment with high moisture). Results showed that amylase supplementation (4 vs. 0 kg/t) increased the contents of small molecular oligosaccharides of extruded corn (*p* < 0.05). Amylase supplementation significantly improved the average daily feed intake, apparent total tract digestibility (ATTD) of dry matter, crude protein, gross energy, crude fat, ash, phosphorus, and calcium, and also increased the activities of jejunal trypsin, α-amylase, lipase, sucrase, maltase, γ-glutamyl transferase and alkaline phosphatase activities, improved the duodenal, jejunal and ileal morphology, and increased the relative mRNA expressions of the *ZO-1*, *OCLN*, *SGLT1*, and *GLUT2* genes in the jejunum (*p* < 0.05), whereas it decreased the contents of isobutyric acid in cecal digesta, as well as acetic acid and isobutyric acid in colonic digesta (*p* < 0.05). Moreover, the linear discriminant analysis effect size (LEfSe) showed that piglets fed extruded corn with added enzymes contained less intestinal pathogenic bacteria, such as *Holdemanella* and *Desulfovibrio*, compared with piglets fed just extruded corn. In summary, the results of the present study indicated that the supplementation of α-amylase during the conditioning and extruding process of corn increased the small molecular oligosaccharide content of corn starch. Moreover, piglets receiving extruded enzyme-added corn had better growth performance, which was associated with the improved intestinal digestive and absorptive function, as well as the intestinal microbiome.

## 1. Introduction

Raw starch, the main energy source of monogastric animals, widely exists in energy feed (corn, wheat, barely, etc.) and can be abruptly stressful in the digestion process of weaning piglets [1,2,3] because weaning stress has been associated with impaired intestinal digestive function [4,5]. Extrusion has been the most common pre-digestion process for raw starch in feed production [6]. The highly ordered structure of starch is destroyed and the gelatinization degree is increased after extrusion [7], which increases the efficiency of enzymatic starch hydrolysis by α-amylase in the small intestine, and further improves the digestibility of starch in weaning piglets [8].

Starch digestion occurs primarily in the piglets’ small intestine, where the pancreatic α-amylase hydrolyzes starch to maltose and larger oligosaccharides, which are further degraded to glucose by brush border membrane oligosaccharidases before absorption [9,10]. Previous research has shown that amylase supplementation in vivo could complement the deficiency of endogenous amylase, improving the digestibility of starch in cereal and enhancing the subsequent growth performance of weaning piglets [11,12,13].

Although the various functions of extrusion processes in vitro or amylase supplementation in vivo have received extensive attention in weaning piglets [1,2,3,4,5,6,7,8,9,10,11,12,13], there are few studies on the application of amylase in vitro during the extrusion of raw corn starch due to the high temperature of the process. However, the optimal reaction temperature of thermostable α-amylase is as high as 105 °C, which implicates the feasibility of in vitro degradation of corn starch via enzymatic hydrolysis during the extrusion process [6,7,8]. Therefore, the present experiment was designed to investigate the effects of extruded corn with added thermostable α-amylase under different moisture conditions on the growth performance, digestive capability, and intestinal microbiome of weaning pigs, which provided new insights into the applications of extruded corn with added enzymes for the pig industry.

## 2. Materials and Methods

All animal procedures associated with this study were approved by the Animal Care and Use committee, Sichuan Agricultural University (Ethic Approval Code: SICAUAC202106-1; Chengdu, China). The primary experiment was conducted at the Animal Experiment Center of Tongwei Co., Ltd., Chengdu, China.

The conditioner (MUTZ1610) and extruder (EXT155G) were provided by Jiangsu Speed Machinery Co., Ltd., Changzhou, China and Beijing Modern Yanggong Technology Development Co., Ltd., Beijing, China, respectively. In addition, the α-amylase (provided per gram of α-amylase units) was provided by Sainuo biological technology Co., Ltd., Baiyin, China.

### 2.1. Experimental Design and Animal Management

A total of 48 twenty-four-d-old pigs (Duroc × Landrace × Yorkshire, weaned at 22 ± 1 d and fed the basal diet for a 3-d adaptation period) with a body weight (BW) of 6.76 ± 0.15 kg were used in a 14-d experiment. At the beginning of the experiment, pigs were assigned to four treatments with six replicates (one male and one female per pen) on the basis of their initial BW and sex in a randomized complete block design. The four dietary treatment groups included: (1) NL (adding 7.5% water before corn extrusion); (2) NH (adding 15.0% water before corn extrusion); (3) PL (adding 7.5% water and 4 kg/t α-amylase before corn extrusion); and (4) PH (adding 15% water and 4 kg/t α-amylase before corn extrusion).

The corn meal was pretreated before conditioning and extruded according to the different treatments. Briefly, NL added water with 7.5% the mass of corn meal, NH added water with 15% the mass of corn meal, PL added water with 7.5% the mass of corn meal and supplemented with 4 kg/t α-amylase, and PH added water with 15% the mass of corn meal and supplemented with 4 kg/t α-amylase; following this, the pre-treated corn meal was prepared under the same conditioning at 90 °C, extruding at 105 °C, cooling at room temperature, and screening at 2.0 mm. The basal diet (Table 1) was formulated based on the NRC (2012) recommendations [14].

All weaning pigs were fed the diets three times per day at 08:00, 14:00, and 20:00 h, provided with water ad libitum, and housed in a temperature- (27 ± 1 °C) and relative-humidity-controlled (55% to 65%) room. All weaning pigs were weighed at the beginning and the end of the experiment after 12 h of fasting, and the average daily gain (ADG), average daily feed intake (ADFI), and feed conversion ratio (F/G) were calculated with feed intake (FI) per pen daily throughout the experiment.

### 2.2. Sampling

The dry basal diet samples and fresh fecal samples collected with a partial collection method per pen after immediate defecation from days 11 to 14 during the trial were collected for chemical analysis. Next, 10 mL of a 10% dilute sulfuric acid solution was added to each 100 g of plastic-bagged fecal sample for corrosion prevention and fixation of excreta nitrogen, which was stored at 20 °C. On day 14, one average BW weaning pig per pen was selected and euthanized with sodium pentobarbital according to a previous study [15], and gut sections were collected from the unfolded abdomen immediately. The complete small intestine was removed, cleaned, and cut into the duodenum, jejunum, and ileum in line with our previous study [16]. Subsequently, approximately 1-cm, 2-cm, and 3-cm segments of proximal duodenum, jejunum, and ileum, respectively, were obtained using the following three steps: immediate isolation, wash with 0.9% physiological saline, and preservation with 10% formaldehyde-phosphate buffer for histological analysis. A 10-cm section of proximal jejunum was gently emptied with gloved hands, carefully flushed with saline, softly placed on an icy surface, and the jejunal mucosa was gently scraped and stored at −80 °C for RNA extraction and activities of digestion-related and absorption-related enzymes. Additionally, for determining gut microbiome and metabolites, the digesta from the middle cecum (10 cm) and middle colon (10 cm) were collected and stored at −80 °C.

### 2.3. Diarrhea

During the experiment from d 1 to d 14, the same person observed and recorded the occurrence of diarrhea on two piglets per pen every morning and evening, and calculated an accumulative diarrhea score per treatment and day to calculate the diarrhea rate according to a previous study [17]. The diarrhea rate was calculated as follows: diarrhea rate (%) = D/(T × 14 d) × 100, in which D = total number of diarrheal pigs per pen and T = number of pigs per pen.

### 2.4. Moisture and Maltooligosaccharides Content in Extruded Corn

The extruded enzyme-added corn samples were analyzed for moisture content (method 930.15; AOAC, 1995) [18]. Moreover, the extruded enzyme-added corn was analyzed using high-performance anion exchange chromatography with pulsed amperometric detection (HPEAC-PAD, Thermo Scientific, Waltham, MA, USA) to detect the maltooligosaccharides content [Glucose (G1), maltose (G2), and maltotetraose through maltoheptaose (G4–G7)] according to the previous study by Ding et al. (2020) [19].

### 2.5. Apparent Digestibility of Nutrients and Enzyme Activities

The fresh feces from days 11 to 14 of each pen were mixed thoroughly with cleaned plastic bags, dried at 65 °C for 72 h, and ground with a small-scale mill to pass through a standard 40-mesh screen. Chromium trioxide (Cr_2_O_3_), as a digestibility indicator, was applied to measure the apparent total tract digestibility (ATTD) of nutrients, with an adaptation period of 4 days. The Cr_2_O_3_ in the diet and fecal samples was analyzed with a Chinese National Standard Method (GB/T 13088-2006) [20]. After the Cr_2_O_3_ analysis, all feed and fecal samples were analyzed for dry matter (method 930.15; AOAC, 1995), ash (method 923.03; AOAC, 1995), crude fat (method 920.39; AOAC, 1995), crude protein (method 990.03; AOAC, 1995), calcium (method 927.02; AOAC, 1995), and phosphorus (method 995.11; AOAC, 1995) [18]. A specific adiabatic oxygen bomb calorimetry (Parr Instrument Co., Moline, IL, USA) was used for the determination of gross energy. The ATTD was calculated as follows: ATTD (%) = {1 − [(A_1_ × F_2_)/(A_2_ × F_1_)]} × 100, in which A_1_ = dietary Cr_2_O_3_ content (% DM), A_2_ = fecal Cr_2_O_3_ content (% DM), F_1_ = dietary nutrient content (% DM), and F_2_ = fecal nutrient content (% DM).

The jejunal sample was per-treated before measuring the activity of digestion and absorption enzymes. Briefly, the jejunal mucosa sample was weighed with pre-cooled saline according to a mass volume ratio of 1:9 (g/mL), and the mixture was homogenized with an utrasonic homogenizer at 4000× *g* for 10 min at 4 °C to collect the supernatant solution. Subsequently, supernatant protein concentration, as well as the activities of trypsin, lipase, α-amylase, β-amylase, lactase, maltase, sucrase, γ-glutamyl transferase, Na^+^-K^+^-ATPase, alkaline phosphatase, and creatine kinase were analyzed using commercial kits (Nanjing Jiancheng Bioengineering Institute, Nanjing, China) according to the manufacturer’s instructions.

### 2.6. Intestinal Morphology

The jejunum morphology was measured as described previously [21]. Briefly, the jejunum preserved in the fixed solution was embedded in paraffin, made into 5-μm slices, flattened with warm water, and baked at 60 °C for 2 h, followed by staining with hematoxylin and eosin, dehydration with alcohol, and sealing with resin glue. An Olympus CK 40 microscope (Olympus Optical Company, Shenzhen, China) was applied to measure villus height and crypt depth. A minimum of 10 complete villi height, and the corresponding depth of 10 crypts, from each jejunal segment were measured.

### 2.7. Real-Time Quantitative PCR

Jejunum mucosal samples (approximately 0.1 g) were homogenized in 1 mL RNAiso Plus reagent (TaKaRa, Dalian, China), and total RNA was extracted and stored at −80 °C according to the instructions. The concentration and quality of total RNA were verified with a nucleic acid protein instrument that measured the corresponding optical density (OD)_260_:OD_280_ ratio, a qualified RNA sample of which should range from 1.8 to 2.0. Meanwhile, the synthesis of cDNA per sample was obtained using a PrimeScript™ reverse transcription reagent kit (TaKaRa, Dalian, China) following the manufacturer’s instructions.

Specific primers for the Sodium-glucose co transporter 1 (*SGLT1*), Glucose transporter 2 (*GLUT2*), Peptide transporter 1 (*PePT1*), Zonula occludens 1 (*ZO-1*), Occludin (*OCLN*), Claudin 1 (*CLDN-1*), and Mucin 1 (*MUC1*) were designed and purchased from Invitrogen (Shanghai, China), which are listed in Table 2. The real-time PCR reactions were performed on a CFX96 Real-Time PCR Detection System (Bio-Rad Laboratories, Inc., Hercules, CA, USA), using SYBR Green PCR reagents (TaKaRa, Dalian, China). After each real-time quantitative PCR test, a melting curve analysis was generated to check and verify the specificity and purity of all PCR products, and β-actin was chosen as the reference gene to normalize cDNA loading. After verifying that the primers amplified with an efficiency of approximately 100%, the results were analyzed using the 2^−ΔΔCt^ method [22].

### 2.8. Gut Microbiome

The QIAamp DNA stool Mini Kit (Qiagen GmbH, Hilden, Germany) was used for the extraction of total genomic DNA from fresh fecal samples. The concentration (purity) and integrity of the extracted genomic DNA were measured using a NanoDrop ND-1000 Spectrophotometer (Nano-Drop Technologies Inc., Wilmington, DE, USA) and electrophoresis on 1% (*w/v*) agarose gels, respectively. Extracted fecal DNA samples were sent to Majorbio Bio-pharm Technology Co., Ltd. (Shanghai, China) to perform amplicon pyrosequencing on the Illumina MiSeq platforms (Illumina Inc., San Diego, CA, USA). The V3–V4 hypervariable region of the 16S rRNA gene was amplified by PCR with primers 338F (5′-ACTCCTACGGGAGGCAGCAG-3′) and 806R (5′-GGACTACHVGGTWTCTAAT-3′). The relative abundance of each operational taxonomic units (OTU) was examined at different taxonomic levels. Diversity within communities (Alpha diversity) calculations and taxonomic community assessments were performed by Mothur 1.30.2 (University of Michigan, Ann Arbor, MI, USA) and Qiime 1.9.1 (National Microbiology Data Center, Beijing, China). Principal coordinates analysis (PCoA) plots were produced using unweighted UniFrac metrics [23]. The linear discriminant analysis (LDA) effect size (LEfSe) method was performed to elucidate the difference among treatments.

### 2.9. Microbial Metabolites Analysis

Approximately 0.7 g of digesta samples from the cecum and colon were used to determine the concentration of volatile fatty acids (VFA) [15]. Briefly, the supernatants of digesta samples were centrifuged at 500× *g* for 10 min after adding 1:1 distilled water, then 2 mL of the supernatant was transferred to a sterile tube and centrifuged at 12,000× *g* for 10 min, after which 1 mL of the supernatant was transferred to a new sterile tube to which 0.2 mL of 25% metaphosphoric acid was added. This was left at room temperature for 30 min and then centrifuged at 12,000× *g* for 10 min. Next, 500 μL of the supernatant was transferred to another sterile tube, to which 500 μL of methanol was added and the mixture was centrifuged at 12,000× *g* for 10 min, with the resulting supernatant being stored at −20 °C until testing. A gas chromatographic system (VARIAN CP-3800, Varian, Palo Alto, CA, USA) was applied for the separation and quantification of the volatile fatty acids (acetic acid, propionic acid and butyric acid, isobutyric acid, valeric acid, and isovaleric acid).

### 2.10. Statistical Analysis

Moisture and maltooligosaccharides content in extruded corn data (*n* = 2) were analyzed by a *t*-test using the statistical program of SAS (SAS Inst. Inc., Cary, NC, USA). Moreover, the other data (*n* = 6) were analyzed by a two-way ANOVA using the Generalized Linear Models procedure of SAS 9.0 software (SAS, Raleigh, NC, USA), which included the effects of water content, enzyme addition, and the interaction between water content and enzyme addition. The results were presented as means and SEM. Duncan’s multiple-range test was used to determine statistical differences among treatment. For significance determination, the data results were significant with *p* < 0.05, and 0.05 < *p* < 0.1 was considered as a tendency.

## 3. Results

### 3.1. Moisture and Maltooligosaccharides Content in Extruded Corn

As shown in Table 3, compared with extruded corn group, the extruded corn with added enzymes group significantly increased the maltose, maltotriose, maltotetraose, maltopentaose, maltohexaose, and maltoheptaose contents in extruded corn (*p* < 0.05).

### 3.2. Growth Performance

As shown in Table 4, no deaths occurred among the four treatments throughout the trial. Enzyme supplementation significantly improved the ADFI of piglets (*p* < 0.05), and tended to have greater ADG (*p* < 0.10); however, there was no significant difference in other growth performance parameters among the four groups (*p* > 0.05).

### 3.3. Apparent Total Tract Digestibility

Table 5 presents the differences in ATTD among the 4 groups. Increasing moisture content tended to decrease the ATTD of crude protein in piglets (*p* < 0.10). Enzyme supplementation significantly improved the ATTD of dry matter, crude protein, gross energy, crude fat, ash, phosphorus, and calcium (*p* < 0.05), as well as tended to increase the ATTD of crude fiber in piglets (*p* < 0.10). In addition, enzyme and moisture treatments had significant interactive effects on the ATTD of ash and phosphorus (*p* < 0.05), and showed a trend for an interactive effect on the ATTD of crude protein nutrients in weaning piglets (*p* < 0.10).

### 3.4. Digestive and Absorptive Enzyme Activities

Table 6 represents the effects of different moisture contents (adding 7.5 vs. 15% water) and enzyme additions (adding 4 kg/t α-amylase or not) to extruded corn on digestive and absorptive enzyme activities in the jejunum of weaning piglets. Enzyme supplementation significantly improved jejunal trypsin, α-amylase, lipase, sucrase, maltase, γ-glutamyl transferase, and alkaline phosphatase activities (*p* < 0.05), whereas enzyme and moisture treatments had a significant interactive effect on γ-glutamyl transferase activity in the jejunum of weaning piglets (*p* < 0.05).

### 3.5. Intestinal Morphology

**Figure 1:**
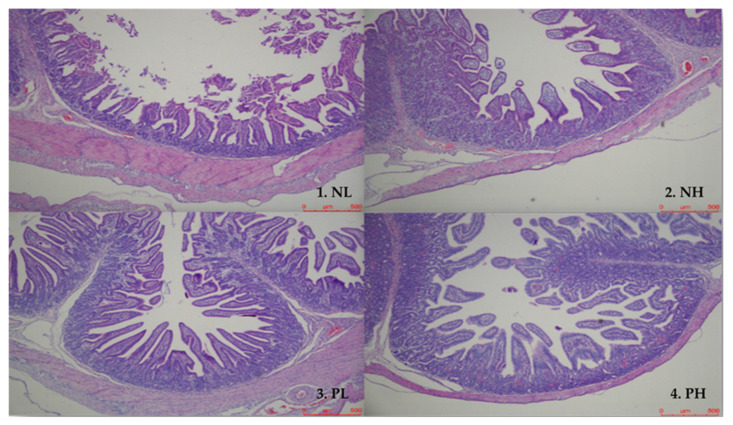
Comparison of jejunal microscopical photographs with histological staining of weaning piglets. NL, adding 7.5% water before corn extrusion; NH, adding 15.0% water before corn extrusion; PL, adding 7.5% water and 4 kg/t α-amylase before corn extrusion; PH, adding 15% water and 4 kg/t α-amylase before corn extrusion.

The status of intestinal morphology in weaning piglets is given in Table 7 and Figure 1. Enzyme treatment significantly increased the duodenal villus height, jejunal villus height, and villus height to crypt depth ratio, as well as the ileal villus height and villus height to crypt depth ratio of weaning piglets (*p* < 0.05), while also showing a trend to reduce the ileal crypt depth of weaning piglets (*p* < 0.10). In addition, there was a trend interaction effect on the jejunal crypt depth between moisture and enzyme treatment (*p* < 0.10).

### 3.6. mRNA Levels of Barrier- and Absorptive-Related Genes

**Figure 2:**
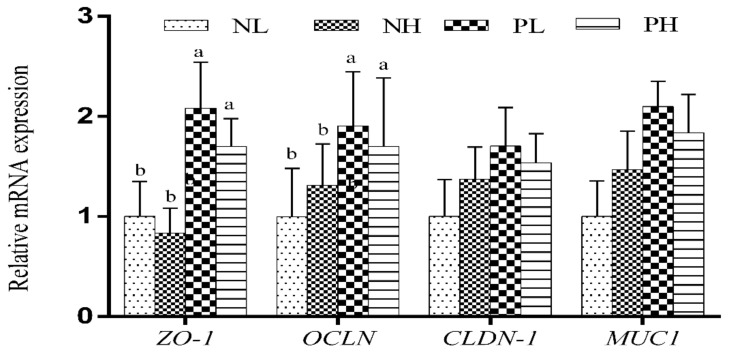
Effects of different moisture contents (adding 7.5 vs. 15% water) and enzyme additions (adding 4 kg/t α-amylase or not) to extruded corn on the mRNA levels of jejunal barrier-function-related genes in weaning piglets. Each column represents the mean expression level with six independent replications. Letters above the bars (a, b) indicate statistical significance (*p* < 0.05) of gene expression among the four treatments. NL, adding 7.5% water before corn extrusion; NH, adding 15.0% water before corn extrusion; PL, adding 7.5% water and 4 kg/t α-amylase before corn extrusion; PH, adding 15% water and 4 kg/t α-amylase before corn extrusion; *ZO-1*, Zonula occludens 1; *OCLN*, Occludin; *CLDN-1*, Claudin 1; *MUC1*, Mucin 1.

The effects of different moisture contents (adding 7.5 vs. 15% water) and enzyme additions (adding 4 kg/t α-amylase or not) to extruded corn on the mRNA levels of jejunal barrier-function-related genes in weaning piglets are presented in Figure 2. The enzyme treatment significantly increased the relative mRNA expressions of the *ZO-1* and *OCLN* genes in the jejunum of weaning piglets (*p* < 0.05). Meanwhile, increasing the moisture content (adding 7.5 vs. 15% water) tended to increase the relative mRNA expression of the *MUC1* gene in the jejunum of weaning piglets (*p* < 0.10).

**Figure 3:**
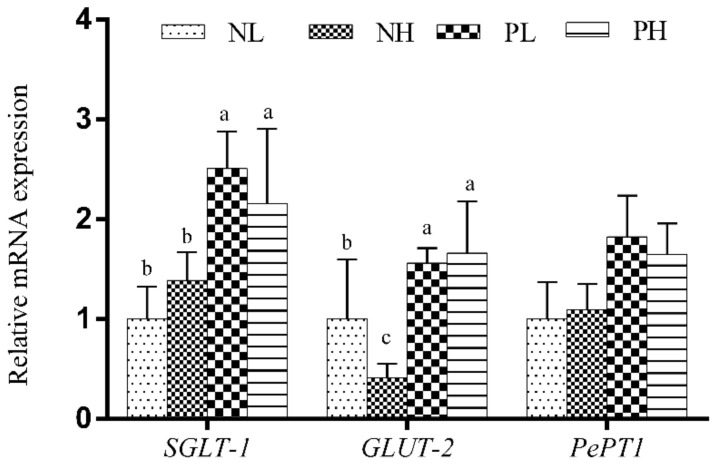
Effects of different moisture contents (adding 7.5 vs. 15% water) and enzyme additions (adding 4 kg/t α-amylase or not) to extruded corn on the mRNA levels of jejunal absorption-related genes in weaning piglets. Each column represents the mean expression level with six independent replications. Letters above the bars (a, b) indicate statistical significance (*p* < 0.05) of genes expression among the four treatments. NL, adding 7.5% water before corn extrusion; NH, adding 15.0% water before corn extrusion; PL, adding 7.5% water and 4 kg/t α-amylase before corn extrusion; PH, adding 15% water and 4 kg/t α-amylase before corn extrusion; *SGLT1*, Sodium-glucose co transporter 1; *GLUT2*, Glucose transporter 2; *PePT1*, Peptide transporter 1.

As shown in Figure 3, the addition of enzymes significantly increased the relative mRNA expressions of the *SGLT-1* and *GLUT-2* genes in the jejunum of weaning piglets (*p* < 0.05), and tended to increase the relative mRNA expression of the *PePT1* gene in the jejunum of weaning piglets (*p* < 0.10).

### 3.7. Gut Microbiome

**Figure 4:**
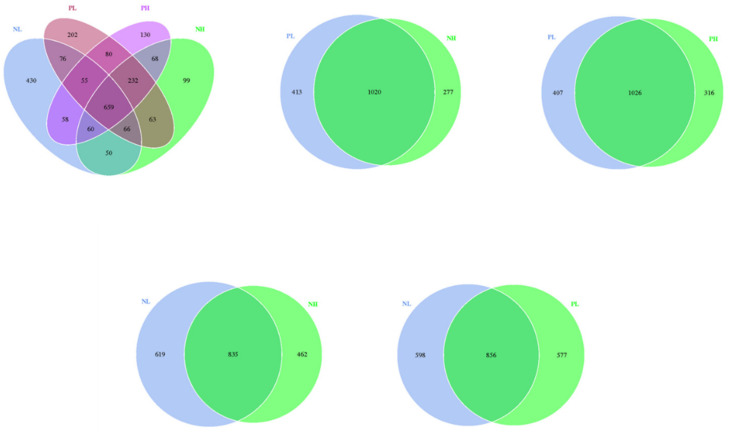
Effects of different moisture contents (adding 7.5 vs. 15% water) and enzyme additions (adding 4 kg/t α-amylase or not) to extruded corn on the gut microbiome structure in weaning piglets. Venn diagram showing the unique and shared OTUs in the different groups. NL, adding 7.5% water before corn extrusion; NH, adding 15.0% water before corn extrusion; PL, adding 7.5% water and 4 kg/t α-amylase before corn extrusion; PH, adding 15% water and 4 kg/t α-amylase before corn extrusion.

From the Venn analysis of OTUs, 430, 202, 130, and 99 unique OTUs were identified in the NL, NH, PL, and PH groups, respectively (Figure 4).

As shown in Table 8, dietary treatments did not markedly affect the observed species, nor the Shannon, Simpson, Chao 1, and ACE indexes (*p* > 0.10).

**Figure 5:**
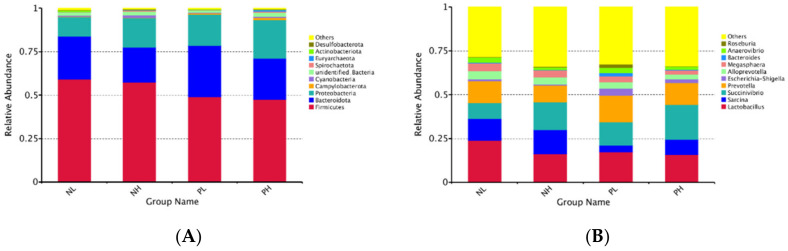
Effects of different moisture contents (adding 7.5 vs. 15% water) and enzyme additions (adding 4 kg/t α-amylase or not) to extruded corn on the relative abundances of gut microbiome phyla (**A**) and genus (**B**) in weaned piglets. The abundance is expressed in terms of a percentage. NL, adding 7.5% water before corn extrusion; NH, adding 15.0% water before corn extrusion; PL, adding 7.5% water and 4 kg/t α-amylase before corn extrusion; PH, adding 15% water and 4 kg/t α-amylase before corn extrusion.

The relative abundances at the phylum level among the treatments are presented in Figure 5A, suggesting that the top nine dominated phyla were Firmicutes, Bacteroidota, Proteobacteria, Campylobacterota, Cyanobacteria, Spirochaetota, Euryarcharota, Actinobacteriota, and Desulfobacterota. The abundance of Firmicutes tended to decrease in the enzyme treatment group (*p* = 0.086). At the genus level, the top 10 dominated genera are shown in Figure 5B, which were *Lactobacillus*, *Sarcina*, *Succinivibrio*, *Prevotella*, *Escherichia-Shigella*, *Alloprevotella*, *Megasphaera*, *Bacteroides*, *Anaerovibrio*, and *Roseburia*.

**Figure 6:**
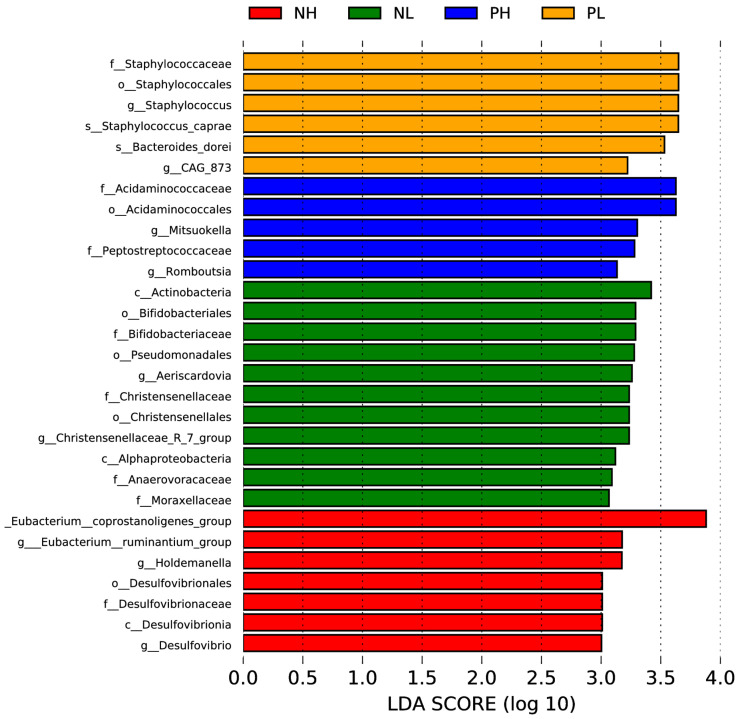
Effects of different moisture contents (adding 7.5 vs. 15% water) and enzyme additions (adding 4 kg/t α-amylase or not) to extruded corn on the phylotypes distribution in weaned piglets. LefSe bar representing differential abundant taxa in pig gut microbiome. NL, adding 7.5% water before corn extrusion; NH, adding 15.0% water before corn extrusion; PL, adding 7.5% water and 4 kg/t α-amylase before corn extrusion; PH, adding 15% water and 4 kg/t α-amylase before corn extrusion.

LEfSe was used to analyze microbial community from the phylum to genus level. The biomarker with statistical difference among the groups was selected with the condition that the set value of the LDA score was 3. As shown in Figure 6, at the genus level, there were significant differences on *Staphylococcus*, *CAG-873*, *Mitsuokella*, *Romboutsia*, *Aeriscardovia*, *Christensenellaceae*-R-7-group, *Eubacterium-ruminantium*-group, *Holdemanella*, and *Desulfovibrio*, in which the LDA values of *Staphylococcus*, *Mitsubishi*, and *Aeriscardovia* ranked first, second, and third, respectively.

### 3.8. Microbial Metabolites

As shown in Table 9, the addition of enzymes significantly reduced the contents of isobutyric acid in the cecal digesta, acetic acid and isobutyric acid in the colonic digesta of weaning piglets (*p* < 0.05), and tended to reduce the content of isovaleric acid in the colonic digesta of weaning piglets (*p* < 0.10). Meanwhile, water significantly decreased the contents of valeric acid in the cecal digesta, butyric acid and valeric acid in the colonic digesta of weaning piglets (*p* < 0.05), and tended to reduce the content of butyric acid in the cecal digesta of weaning piglets (*p* < 0.10).

## 4. Discussion

Previous studies have shown that the moisture content of corn meal during conditioning could affect the maltooligosaccharides content in extruded corn [24,25]. The present study found that enzyme treatment increased the content of small molecular sugars, especially the oligosaccharides composed of 6–7 glucose molecules, which was consistent with the previous study [26,27] that reported that the addition of amylase during the conditioning and extruding process could increase the content of maltooligosaccharides.

The moisture content of raw materials, as a medium of α-amylase action, has a synergistic effect on enzymatic hydrolysis, which plays a key role in the extrusion with added enzymes [28]. A low moisture content of raw materials could be detrimental to the enzymatic reaction, and an excessive moisture content of raw materials made against the extrusion effect and subsequent processing [29,30]. In the present study, it was found that enzyme treatment significantly increased animal feed intake and further improved the daily weight gain of animals. However, the increase of moisture content from 7.5% to 15% had no significant effect on animal growth performance, and there was no interaction effect between the moisture and enzyme treatments.

During the post-weaning period, piglets often suffer from social (maternal and littermate separation), environmental (transportation), and dietary (abrupt change in diets) stressors [31,32]. Weaning stress was associated with destroyed intestinal morphology [33], impaired intestinal barrier, lower activities of epithelial brush border enzyme, decreased digestibility of nutrients, and a subsequently reduced performance [34,35,36]. The present study found that the enzyme treatment improved the intestinal morphology of weaning piglets. We supposed that positive results observed in the study herein may be owing to the following reasons: the low feed intake during the period of weaning resulted in a decrease in villus height and an increase in crypt depth [37]; and extrusions with added enzymes significantly increased the feed intakes of weaning pigs and improved the digestibility of corn starch in the first two weeks, as it was reported that the high feed intakes after weaning could improve the morphology of the small intestine in weaning piglets [33].

In the present study, it was found that enzyme treatment significantly improved the ATTD of nutrients in weaning piglets, while moisture content had no significant effect. This was consistent with growth performance, indicating that the pre-digestion effect of α-amylase on corn starch during extrusion was the main reason to improve the nutrients’ digestibility. The enzyme activity in the digestive tract was considered to be a key factor affecting intestinal health and nutrient digestibility [38]. In addition, many previous studies have shown that the exogenous addition of amylase or improvement of starch gelatinization can promote the activity of digestive enzymes in weaning piglets [39,40]. The present study found that the enzymatic hydrolysis of starch in the process of extrusion in vitro can significantly improve the jejunal trypsin and α-amylase, lipase, sucrase, maltase, γ-glutamine transferase, and alkaline phosphatase activities. Recent studies showed that *SGLT1*, *GLUT2*, and *PePT1* were extensively located at the intestinal mucosa, where they serve as the main transporters, respectively, for glucose, glucose, and amino acids [41]. We found that enzyme treatment significantly increased the relative mRNA expressions of the *SGLT-1* and *GLUT-2* genes in the jejunum of weaning piglets, which was consistent with the results reported by other studies, where starch that was hydrolyzed in vitro by α-amylase could significantly increase the mRNA expressions of *SGLT-1* and *GLUT-2* genes in IPEC-J2 cell [42].

The integrity of the intestinal barrier played an important role in the proper functioning of the epithelial cells, which could reduce the increment of pathogenic bacteria [43]. However, weaning stress contributed to impaired intestinal barrier function and enhanced intestinal permeability [36]. The intestinal tight junction protein family (*CLDN*, *OCLN*, *ZO*, etc.) determined endothelial and epithelial paracellular barrier functions, which prevented the paracellular diffusion of intestinal bacteria and other antigens across the epithelium [44,45]. The present study found that the enzyme treatment significantly increased the relative mRNA expressions of the *ZO-1* and *OCLN* genes in the jejunum of weaning piglets, a possible reason for which might owned to the improvement of small molecular oligosaccharides by α-amylase action during the conditioning and extruding process, which may contain some oligosaccharides with intestinal regulation function, such as Isomaltooligosaccharides. It was found that the addition of Isomaltooligosaccharide could increase the relative mRNA expression of the *OCLN* gene in intestinal epithelial cells, increasing the intestinal barrier function of weaning piglets [46].

The intestine microorganisms are associated with digestive and absorptive functions, as well as the subsequent gut health [47]. In our study, 16S rRNA sequencing was used to investigate the gut microbiome responses to moisture contents (adding 7.5 vs. 15% water) and enzyme additions (adding 4 kg/t α-amylase or not) to extruded corn in weaning piglets. Our results showed that moisture content and enzyme treatment reduced the number of OTUs of the fecal microbiome, but there was no significant difference in the results of the alpha diversity analysis of the fecal microbiome. At the phylum level, the abundance of Firmicutes tended to decrease in the enzyme treatment group, suggesting that the enzyme treatment may reduce the polysaccharide fermentation of hindgut flora, and further reducing the production of volatile fatty acids (VFAs).

In the present study, piglets fed NL diets increased *Aeriscardovia* and *Christensenlace*-r-7-group. The previous study found that the *Christensenlace*-r-7-group was significantly negatively correlated with metabolic diseases such as body index and inflammation [48]. *Aeriscardovia* could synthesize a variety of enzymes, amino acids, and vitamins by itself and metabolize acetic acid, lactic acid, and so on [49]. The dominant bacteria in the NH group were *Holdemanella* and *Desulfovibrio*. *Holdemanella* was a harmful bacterium associated with intestinal inflammation [50], and studies have shown that *Desulfovibrio* belong to desulfobacterate, and its role might be related to the occurrence of some inflammatory intestinal diseases and colon cancer [51]. The dominant bacteria in the PL group were *Staphylococcus* and *Cag-873*. *Staphylococcus* is a gram-positive cocci, which might have a negative effect on intestinal health [52]. *Cag-873* was a genus of Bacteroidetes, which was related to hindgut energy homeostasis and had the ability to degrade cellulose, hemicellulose, and lignin [53]. In addition, the dominant bacteria in the PH group were *Mitsuokella* and *Romboutsia*, where *Mitsuokella* mediates the positive regulation of cell-growth-related genes through the metabolism of acetic acid, and may inhibit the growth of intestinal *Salmonella* [54].

The change of gut microbiome composition contributes to the variation in microbial metabolites. The VFAs are not only positive in providing energy for intestinal epithelial cells, but also promote the formation of cells, improving intestinal morphology [55]. Among VFAs, acetic acid, propionic acid, and butyric acid are the most important VFAs, and the change of their composition and proportion can, in turn, affect the change of the gut microbiome. Butyric acid was the energy substrate of colonic epithelial cells and can inhibit colonic cell apoptosis [56]. Acetic acid had the highest concentration among VFAs, accounting for about 60%, followed by propionic acid and butyric acid; in addition, acetic acid is a potential precursor of cholesterol and fatty acids, which can be used as a substrate for the de novo synthesis of fatty acids [57]. While propionic acid could participate in gluconeogenesis, and valeric acid and isobutyric acid are the products of bacterial fermentation proteins, marking the catabolism of intestinal proteins [58]. Meanwhile, isobutyric acid could enhance intestinal permeability, which may have a negative impact on intestinal health [59]. The present study found that the enzyme treatment significantly reduced the contents of isobutyric acid in the cecal digesta, as well as acetic acid and isobutyric acid in the colonic digesta of weaning piglets, and tended to reduce the total VFAs in the colon digesta; a possible reason these results might be related to the higher digestibility of starch and protein, subsequently lowering the fermentation substrate of hindgut flora when supplemented with α-amylase.

## 5. Conclusions

In summary, the results of the present study showed that supplementation of α-amylase during the conditioning and extruding process of corn meal increased the proportion of small molecular oligosaccharides. Piglets receiving extruded corn with added enzymes had better growth performance, which was associated with improved intestinal digestive function and intestinal microbiome. Therefore, our results suggest that extruded corn with added enzymes could be a potential feeding processing method for enhancing the health and growth of weaning pigs.

## Figures and Tables

**Table 1:** Compositions and nutrient contents of the experiment basal diets ^1^ (air dry basis, %).

Item	
Ingredients, %	
Extruded corn	66.485
Whole-fat milk powder	15.00
Soy protein isolate	10.00
Coconut oil	5.00
Calcium carbonate	0.45
Dicalcium phosphate	0.90
Choline chloride	0.05
L-Lysine HCl (98.5%)	0.64
DL-Methionine	0.27
L-Threonine	0.245
L-Tryptophan	0.075
L-Valine	0.185
L-Arginine	0.170
NaCl	0.20
Vitamin premix ^2^	0.03
Mineral premix ^3^	0.30
Total	100
Calculated Nutrients ^4^	
DE (Mcal/kg)	3.69
CP (%)	18.34
Ca (%)	0.49
TP (%)	0.51
AP (%)	0.30
STTDP (%)	0.35
SID-Lysine	1.36
SID-Met	0.53
SID-Met + Cys	0.75
SID-Thr	0.81
SID-Trp	0.25

^1^ DE, digestive energy; CP, crude protein; Ca, calcium; TP, total phosphorus; AP, available phosphorus; STTDP, standardized total tract digestibility phosphorus; SID, Standardized ileal digestibility. ^2^ Provided per kilogram of diet: vitamin A, 9000 IU; vitamin D_3_, 3000 IU; vitamin E, 20 IU; vitamin K_3_, 3.0 mg; vitamin B_1_, 1.5 mg; vitamin B_2_, 4.0 mg; vitamin B_6_, 3.0 mg; vitamin B_12_, 0.2 mg; nicotinic acid, 30 mg; pantothenic acid, 15 mg; folic acid, 0.75 mg; and biotin, 0.1 mg. ^3^ Provided per kilogram of diet: 120 mg Fe (FeSO_4_·7 H_2_O); 20 mg Cu (CuSO_4_·5H_2_O); 120 mg Zn (ZnSO_4_·7H_2_O); 15 mg Mn (MnSO_4_·H_2_O); 0.4 mg Se (Na_2_SeO_3_·5H_2_O); and 0.3 mg I (KI). ^4^ Values are calculated.

**Table 2:** Sequence of primers used for the real-time quantitative PCR analysis ^1^.

Genes ^1^	Primer Sequences (5′–3′) ^2^	Size (bp)	A_T_^3^, °C	Accession Number
*β-actin*	F: TCTGGCACCACACCTTCT	114	60	DQ178122
	R: TGATCTGGGTCATCTTCTCAC			
*SGLT1*	F: GCAACAGCAAAGAGGAGCGTAT	137	60	NM_001164021.1
	R: GCCACAAAACAGGTCATAGGTC			
*GLUT2*	F: GACACGTTTTGGGTGTTCCG	149	58	NM_001097417.1
	R: GAGGCTAG-CAGATGCCGTAG			
*PePT1*	F: GCCAAAGTCGTCAAGTGC	100	58.5	NM_214347
	R: GGTCAAACAAAGCCCAGA			
*ZO-1*	F: CTGAGGGAATTGGGCAGGAA	105	60	XM_013993251.1
	R: TCACCAAAGGACTCAGCAGG			
*OCLN*	F: CAGGTGCACCCTCCAGATTG	110	60	XM_001163647.2
	R: GGACTTTCAAGAGGCCTGGAT			
*CLDN-1*	F: GCCACAGCAAGGTATGGTAAC	140	62	FJ873109.1
	R: AGTAGGGCACCTCCCAGAAG			
*MUC1*	F: GTGCCGCTGCCCACAACCTG	141	61	XM_001926883.5
	R: AGCCGGGTACCCCAGACCCA			

^1^*SGLT1*, Sodium-glucose co transporter 1; *GLUT2*, Glucose transporter 2; *PePT1*, Peptide transporter 1; *ZO-1*, Zonula occludens 1; *OCLN*, Occludin; *CLDN-1*, Claudin 1; *MUC1*, Mucin 1. ^2^ F = forward primer; R = reverse primer. ^3^ A_T_ = annealing temperature.

**Table 3:** Effects of different enzymes (adding 4 kg/t α-amylase or not) being added to extruded corn on the moisture content and maltooligosaccharides contents of extruded corn ^1^.

Item	Extruded Corn	Extruded Enzyme-Added Corn	SEM ^2^	*p*-Value
Moisture content, %	10.56	11.34	0.14	0.675
Glucose, unit	8.53	8.89	0.10	0.871
Maltose, unit	4.46 ^b^	6.42 ^a^	0.45	<0.01
Maltotriose, unit	0.43 ^b^	3.28 ^a^	0.32	<0.01
Maltotetraose, unit	0.24 ^b^	3.41 ^a^	0.31	<0.01
Maltopentaose, unit	0.16 ^b^	2.23 ^a^	0.24	<0.01
Maltohexaose, unit	0.15 ^b^	2.62 ^a^	0.17	<0.01
Maltoheptaose, unit	0.53 ^b^	3.46 ^a^	0.45	<0.01

^a,b^ Means in a row with different letter differ (*p* < 0.05). ^1^ Means represent *n* = 2. ^2^ Standard error of the means.

**Table 4:** Effects of different moisture contents (adding 7.5 vs. 15% water) and enzyme additions (adding 4 kg/t α-amylase or not) to extruded corn on the growth performance and diarrhea rate in weaning piglets ^1^.

Item ^2^	Enzyme−		Enzyme+		SEM ^3^	*p*-Value ^4^		
	7.5%	15%	7.5%	15%		p *	*p ^+^*	*p ^#^*
Initial weight, kg	6.76	6.74	6.78	6.77	0.15			
Final weight, kg	11.16	10.88	11.65	11.33	0.28	0.46	0.25	0.96
ADG, g/d	314.64	295.60	347.98	325.95	12.46	0.26	0.09	0.93
ADFI, g/d	473.15 ^b^	451.19 ^b^	522.80 ^a^	494.05 ^a^	15.61	0.26	0.04	0.88
F/G	1.51	1.54	1.50	1.52	0.03	0.57	0.73	0.83
Diarrhea rate	0.11	0.11	0.09	0.11	0.01	0.41	0.62	0.62

^a,b^ Means different letters in a row differ (*p* < 0.05). ^1^ Means represent *n* = 6. ^2^ ADG, average daily gain; ADFI, average daily feed intake; F/G, feed conversion ratio. ^3^ Standard error of the means. ^4^ *p* * Means moisture effect, *p* ^+^ Means enzyme effect, and *p* ^#^ Means water × enzyme interaction effect.

**Table 5:** Effects of different moisture contents (adding 7.5 vs. 15% water) and enzyme additions (adding 4 kg/t α-amylase or not) to extruded corn on the apparent total tract digestibility in weaning piglets ^1^.

Item	Enzyme−		Enzyme+		SEM ^2^	*p*-Value ^3^		
	7.5%	15%	7.5%	15%		p *	*p ^+^*	*p ^#^*
Dry matter	88.65 ^b^	87.67 ^b^	89.90 ^a^	90.11 ^a^	0.42	0.37	<0.01	0.18
Crude protein	82.86 ^b^	80.84 ^b^	86.08 ^a^	86.23 ^a^	0.53	0.09	<0.01	0.06
Gross energy	88.46 ^b^	87.54 ^b^	89.75 ^a^	90.09 ^a^	0.39	0.47	<0.01	0.12
Crude fiber	50.70	49.88	59.65	52.26	3.26	0.22	0.09	0.33
Crude fat	69.82 ^b^	66.78 ^b^	73.87 ^a^	74.58 ^a^	1.47	0.44	0.01	0.22
Ash	48.37 ^b^	39.47 ^b^	55.37 ^a^	60.50 ^a^	2.63	0.48	<0.01	0.02
Phosphorus	52.14 ^b^	46.17 ^b^	59.29 ^a^	63.24 ^a^	2.25	0.66	<0.01	0.04
Calcium	54.41 ^b^	48.70 ^b^	64.23 ^a^	66.24 ^a^	2.67	0.50	<0.01	0.16

^a,b^ Means different letters in a row differ (*p* < 0.05). ^1^ Means represent *n* = 6. ^2^ Standard error of the means. ^3^ *p* * Means moisture effect, *p* ^+^ Means enzyme effect, and *p* ^#^ Means water × enzyme interaction effect.

**Table 6:** Effects of different moisture contents (adding 7.5 vs. 15% water) and enzyme additions (adding 4 kg/t α-amylase or not) to extruded corn on the digestive and absorptive enzyme activities in the jejunum of weaning piglets ^1^.

Item	Enzyme−		Enzyme+		SEM ^2^	*p*-Value ^3^		
	7.5%	15%	7.5%	15%		p *	*p ^+^*	*p ^#^*
Trypsin, U/mgprot	2168.48 ^b^	1670.54 ^c^	2889.76 ^a^	2672.38 ^a^	146.08	0.02	<0.01	0.35
α-amylase, U/mgprot	276.03 ^b^	260.21 ^b^	421.73 ^a^	391.03 ^a^	34.25	0.50	0.01	0.83
β-amylase, U/mgprot	15.16	16.07	13.48	12.44	2.43	0.29	0.98	0.69
Lipase, U/gprot	248.89 ^b^	243.55 ^b^	418.13 ^a^	401.99 ^a^	52.63	0.84	<0.01	0.92
Sucrase, U/mgprot	109.67 ^c^	141.12 ^b^	162.66 ^a^	157.65 ^a^	15.56	0.41	0.03	0.26
Maltase, U/gprot	285.53 ^b^	303.54 ^b^	399.00 ^a^	396.67 ^a^	35.84	0.83	<0.01	0.78
Lactase, U/gprot	144.37	143.23	139.09	123.85	43.69	0.85	0.78	0.87
γ-glutamyl transferase, U/L	83.30 ^b^	125.65 ^a^	125.80 ^a^	122.07 ^a^	10.66	0.19	0.03	0.02
Na^+^-K^+^-ATPase, U/mgprot	24.37	14.25	21.94	29.30	5.16	0.79	0.24	0.11
Alkaline phosphatase, King U/gprot	110.12 ^b^	99.37 ^b^	125.11 ^a^	129.59 ^a^	10.41	0.77	0.04	0.47
Creatine kinase, U/mgprot	21.24	22.99	22.09	20.33	1.42	0.21	0.68	0.34

^a,b,c^ Means different letters in a row differ t (*p* < 0.05). ^1^ Means represent *n* = 6. ^2^ Standard error of the means. ^3^ *p* * Means water effect, *p* ^+^ Means enzyme effect, and *p* ^#^ Means water × enzyme interaction effect.

**Table 7:** Effects of different moisture contents (adding 7.5 vs. 15% water) and enzyme additions (adding 4 kg/t α-amylase or not) to extruded corn on the intestinal morphology of weaning piglets ^1^.

Item	Enzyme−		Enzyme+		SEM ^2^	*p*-Value ^3^		
	7.5%	15%	7.5%	15%		p *	*p ^+^*	*p ^#^*
Duodenum								
Villus height, μm	647.87 ^c^	617.47 ^c^	815.40 ^a^	705.50 ^b^	57.68	0.24	0.04	0.50
Crypt depth, μm	306.64	297.35	323.91	308.94	29.80	0.69	0.63	0.93
Villus to crypt ratio	2.26	2.11	2.70	2.31	0.25	0.30	0.22	0.64
Jejunum								
Villus height, μm	434.29 ^b^	436.27 ^b^	555.31 ^a^	527.65 ^a^	48.83	0.80	0.04	0.77
Crypt depth, μm	185.34	225.15	216.14	179.24	20.14	0.94	0.71	0.07
Villus to crypt ratio	2.36 ^b^	2.06 ^b^	2.83 ^a^	2.94 ^a^	0.29	0.74	0.03	0.50
Ileum								
Villus height, μm	412.29 ^b^	447.57 ^b^	490.44 ^a^	465.72 ^a,b^	22.92	0.82	0.04	0.21
Crypt depth, μm	186.16	207.84	157.97	157.46	20.40	0.61	0.06	0.59
Villus to crypt ratio	2.37 ^b^	2.33 ^b^	3.15 ^a^	3.07 ^a^	0.25	0.80	<0.01	0.94

^a,b,c^ Means different letters in a row differ (*p* < 0.05). ^1^ Means represent *n* = 6. ^2^ Standard error of the means. ^3^ *p* * Means moisture effect, *p* ^+^ Means enzyme effect, and *p* ^#^ Means water × enzyme interaction effect.

**Table 8:** The alpha diversity in the fecal microbiome of weaning piglets ^1^.

Item	Enzyme−		Enzyme+		SEM ^2^	*p*-Value ^3^		
	7.5%	15%	7.5%	15%		p *	*p ^+^*	*p ^#^*
Observed species	729.80	643.50	624.50	651.17	97.29	0.466	0.238	0.174
Shannon	5.20	5.32	5.34	5.36	0.49	0.758	0.696	0.826
Simpson	0.91	0.92	0.93	0.92	0.03	0.827	0.488	0.488
Chao 1	793.47	702.61	684.37	707.10	107.59	0.456	0.257	0.220
ACE	818.87	732.28	698.86	716.37	114.18	0.473	0.166	0.284

^1^ Means represent *n* = 6. ^2^ Standard error of the means. ^3^ *p* * Means moisture effect, *p* ^+^ Means enzyme effect, and *p* ^#^ Means water × enzyme interaction effect.

**Table 9:** Effects of different moisture contents (adding 7.5 vs. 15% water) and enzyme additions (adding 4 kg/t α-amylase or not) to extruded corn on intestinal microbial metabolites (μmol/g of wet digesta) in the cecal and colonic digesta of weaning piglets ^1^.

Item	Enzyme−		Enzyme+		SEM ^3^	*p*-Value ^4^		
	7.5%	15%	7.5%	15%		p *	*p ^+^*	*p ^#^*
Cecal digesta								
Acetic acid	4.11	3.91	3.74	3.71	0.55	0.606	0.226	0.722
Propanoic acid	2.46	2.41	2.52	2.36	0.53	0.638	0.989	0.805
Butyric acid	0.27	0.19	0.23	0.20	0.08	0.091	0.699	0.489
Isobutyric acid	0.12 ^a^	0.10 ^a,b^	0.08 ^b^	0.07 ^b^	0.04	0.197	0.036	0.644
Valeric acid	0.39 ^a^	0.20 ^b^	0.32 ^a^	0.23 ^b^	0.16	0.041	0.740	0.438
Isovaleric acid	0.10	0.08	0.08	0.06	0.04	0.276	0.254	0.899
TVFAs ^2^	7.47	6.89	6.99	6.63	1.10	0.326	0.439	0.816
Colonic digesta								
Acetic acid	4.06	3.80	3.47	3.39	0.50	0.376	0.013	0.614
Propanoic acid	2.37	2.16	2.28	2.16	0.41	0.351	0.789	0.802
Butyric acid	0.23 ^a^	0.19 ^b^	0.23 ^a^	0.19 ^b^	0.04	0.016	0.997	0.844
Isobutyric acid	0.12 ^a^	0.12 ^a^	0.09 ^b^	0.09 ^b^	0.03	0.693	0.013	0.968
Valeric acid	0.35 ^a^	0.23 ^b^	0.32 ^a^	0.25 ^b^	0.10	0.030	0.958	0.531
Isovaleric acid	0.11	0.11	0.09	0.09	0.03	0.517	0.074	0.977
TVFAs ^2^	7.25	6.60	6.48	6.17	0.89	0.184	0.098	0.635

^a,b^ Means in a row with different letter differ (*p* < 0.05). ^1^ Means represent 6 pens with 2 pigs per pen (*n* = 6). ^2^ TVFAs, total volatile fatty acids. ^3^ Standard error of the means. ^4^ *p* * Means moisture effect, *p* ^+^ Means enzyme effect, and *p* ^#^ Means water × enzyme interaction effect.

## Data Availability

The datasets used to support the findings of this study are available from the corresponding author upon request.
